# Validation of Non-Invasive Hemoglobin Measurement and Its Utility for Anemia Screening in Pregnancy: A Cross-Sectional Diagnostic Accuracy Study

**DOI:** 10.3390/diagnostics16183005

**Published:** 2026-09-17

**Authors:** Suchaya Luewan, Mattanaporn Wongyai, Theera Tongsong

**Affiliations:** 1 Department of Obstetrics and Gynecology, Faculty of Medicine, Chiang Mai University, Chiang Mai 50200, Thailand; 2 Fetal Center, Faculty of Medicine, Chiang Mai University, Chiang Mai 50200, Thailand

**Keywords:** non-invasive hemoglobin measurement, Masimo Rad-67, SpHb, anemia, pregnancy, screening, pulse CO-oximetry, perfusion index

## Abstract

**Objective:** To evaluate the agreement between non-invasive hemoglobin (SpHb) and venous complete blood count (CBC) in measurements of Hb in pregnant women and to compare the diagnostic performance in detecting anemia. **Methods:** A prospective cross-sectional study enrolled 660 Thai pregnant women, with 220 participants in each trimester. SpHb was measured at the thumb (tHb) and index finger (fHb) using the Masimo Rad-67, while venous CBC hemoglobin (CBC-Hb) served as the reference. Agreement between SpHb and CBC-Hb was evaluated using Bland–Altman analysis and the intraclass correlation coefficient (ICC). Potential factors influencing SpHb measurement accuracy were identified using generalized linear models, and the diagnostic performance of the resulting predictive models was subsequently evaluated. **Results:** The mean difference between SpHb and CBC-Hb was significant, with SpHb consistently overestimating hemoglobin concentrations across all trimesters. Consequently, SpHb demonstrated very low sensitivity (2.1% for tHb and 3.2% for fHb) but high specificity for detecting maternal anemia. Agreement with CBC-Hb was poor for absolute measurements (ICC = 0.221–0.278) but moderate for consistency (ICC = 0.615–0.650), indicating moderate precision but poor accuracy. Although optimized SpHb cut-offs substantially improved diagnostic performance, the two methods remained non-interchangeable. Measurement accuracy was significantly influenced by gestational trimester, maternal BMI, and perfusion index, underscoring the need for pregnancy- and ethnicity-specific calibration algorithms. **Conclusions:** Although adjustment models improved the diagnostic performance of SpHb for detecting maternal anemia, the substantial positive systematic bias and only moderate agreement suggest that SpHb should not replace CBC-Hb for hemoglobin measurement without further calibration. With these refinements, non-invasive Hb measurement may become a practical and effective screening approach in pregnancy.

## 1. Introduction

Anemia in pregnancy is a major global public health problem, affecting 35.5% of pregnant women aged 15–49 years worldwide according to the World Health Organization 2023 estimates [1]. In Thailand, the prevalence is approximately 39%, comparable to the global burden [2,3]. Iron deficiency accounts for about half of all cases globally, while thalassemia trait, vitamin B12 deficiency, and folate deficiency are also important causes, particularly in Southeast Asia [4].

Maternal anemia is associated with adverse maternal outcomes, including preeclampsia, heart failure, postpartum hemorrhage, and postpartum depression, as well as fetal and neonatal complications such as fetal growth restriction, low birth weight, preterm birth, and impaired neurodevelopment with lasting effects on cognitive function [4]. Because these developmental deficits may not be fully reversible despite childhood iron supplementation, early detection and timely treatment during pregnancy are essential.

The standard screening method for anemia in pregnancy is the complete blood count (CBC), which requires venipuncture, laboratory infrastructure, trained personnel, and processing time. In Thailand, all pregnant women receive CBC screening at their first antenatal visit along with preliminary thalassemia carrier screening. However, the requirement for venipuncture and laboratory processing can be a barrier in resource-limited settings, and repeated testing throughout pregnancy may be challenging to implement systematically.

Non-invasive hemoglobin measurement using pulse CO-oximetry has emerged as a potential screening tool. The Masimo Rad-67 Pulse CO-Oximeter^®^ (Masimo Corporation, Irvine, CA, USA) estimates hemoglobin concentration (SpHb) spectrophotometrically without blood sampling and has shown variable performance across perioperative, emergency, and pediatric settings [5,6,7,8,9,10,11,12,13]. Studies in pregnant women have likewise reported inconsistent findings, ranging from acceptable agreement with laboratory hemoglobin to significant bias and poor accuracy for anemia detection [14,15,16,17]. Pregnancy-related physiological changes, including plasma volume expansion, altered peripheral perfusion, and tissue composition, together with factors such as perfusion index, skin pigmentation, nail polish, and ambient temperature, may further influence SpHb accuracy [18]. Additionally, ethnic differences may influence device calibration, as the biophysical characteristics of peripheral perfusion can vary across populations.

Despite the potential advantages of non-invasive hemoglobin measurement for antenatal anemia screening, evidence regarding its diagnostic accuracy across different trimesters of pregnancy in Southeast Asian populations remains limited. The objectives of this study were primarily (1) to evaluate the agreement between non-invasive hemoglobin (SpHb) measured using the Masimo Rad-67 and venous complete blood count-derived hemoglobin (CBC-Hb) in pregnant women and (2) to assess the diagnostic performance of SpHb for detecting maternal anemia using CBC-Hb as the reference standard. The secondary objective was to determine whether biophysical parameters, including body mass index (BMI), gestational trimester, pulse rate, oxygen saturation, and perfusion index, influenced the accuracy of non-invasive hemoglobin (SpHb) measurements.

## 2. Patients and Methods

This was a prospective cross-sectional diagnostic accuracy study conducted at two sites in Chiang Mai, Thailand: Maharaj Nakorn Chiang Mai Hospital (a tertiary university hospital) and Sansai Hospital (a secondary community hospital), between July 2024 and February 2025. The study protocol was approved by the Research Ethics Committee of the Faculty of Medicine, Chiang Mai University (approval number 263/2567, Study Code: OBG-2567-0315), and all participants provided written informed consent. Pregnant women aged > 20 years with singleton pregnancies attending routine antenatal care were invited to participate. Eligible participants were required to have a confirmed gestational age based on the last menstrual period or first- or early second-trimester ultrasound. Exclusion criteria included: (1) wounds, skin lesions, or desquamation of the thumb or index finger that could interfere with sensor signal acquisition, and (2) a thumb or index finger size incompatible with the measurement sensor. Participants who refused venipuncture or wished to withdraw were excluded from the analysis. Sample size was calculated and assuming sensitivity and specificity of approximately 80%, a minimum of 196 participants per trimester was required. We aimed to recruit 220 participants per trimester (660 total) to account for potential exclusions and missing data. Participants were classified into three groups based on gestational age at enrollment: first trimester, gestational age ≤ 13^+6^ weeks; second trimester, gestational age 14^+0^ to 27^+6^ weeks; and third trimester, gestational age ≥ 28^+0^ weeks.

After obtaining informed consent, demographic and clinical data were collected, including maternal age, gestational age, gravidity, parity, pre-pregnancy weight and height (for BMI calculation), and relevant medical history.

Non-invasive hemoglobin measurement was performed using the Masimo Rad-67 Pulse CO-Oximeter^®^ with the Rainbow^®^ sensor (Masimo Corporation, Irvine, CA, USA). Measurements were obtained from both the thumb (tHb) and index finger (fHb) of the same hand. Both sites were selected because sensor placement and local peripheral perfusion may affect signal acquisition and SpHb measurement stability; therefore, site-specific measurements were compared with CBC-Hb to assess whether measurement performance differed by measurement site [19]. The sensor was applied according to the manufacturer’s instructions, and measurements were recorded after signal quality indicators showed adequate perfusion and stable readings (typically 60 s). The device displays SpHb (g/dL), oxygen saturation (SpO_2_), perfusion index (PI), and pulse rate. All measurements were performed by trained research nurses using standardized protocols. The venous blood samples were collected by standard venipuncture technique into EDTA tubes for CBC analysis in the same day after SpHb measurement. Anemia was defined according to WHO criteria adapted for pregnancy: hemoglobin < 11.0 g/dL in the first trimester (gestational age < 14 weeks), hemoglobin < 10.5 g/dL in the second trimester (gestational age 14^+0^–27^+6^ weeks), and hemoglobin < 11.0 g/dL in the third trimester (gestational age ≥ 28^+0^ weeks).

Statistical Analysis: Continuous variables were summarized as mean ± standard deviation (SD) or median (interquartile range [IQR]), as appropriate according to data distribution, while categorical variables were presented as frequencies and percentages. Paired *t*-tests were used to compare SpHb and CBC-Hb measurements. The correlation between SpHb and CBC-Hb was assessed using Pearson’s correlation coefficient, whereas agreement was evaluated using Bland–Altman analysis and the intraclass correlation coefficient (ICC). The diagnostic performance of SpHb in detecting maternal anemia was assessed using receiver operating characteristic (ROC) curve analysis. Diagnostic accuracy measures, including sensitivity, specificity, positive predictive value, negative predictive value, positive and negative likelihood ratio were calculated, and area under the ROC curve (AUC), were also calculated. For the secondary objective, factors influencing the accuracy of SpHb measurements were identified using generalized linear models, and the diagnostic performance of the adjusted models for detecting maternal anemia was assessed and presented as an apparent performance of the same dataset using the optimal probability cut-off based on Yooden’s index. Statistical significance was defined as a two-sided *p* value < 0.05. Statistical analyses were performed using SPSS Statistics version 26.0 (IBM Corp., Armonk, NY, USA).

## 3. Results

A total of 669 pregnant women were enrolled (223, 221, and 225 in the first, second, and third trimesters, respectively). After excluding nine participants because of incomplete data or technical measurement issues, 660 women (220 per trimester) were included in the analysis. The overall median age was 30 years (IQR 26–34), and the median BMI was 22.72 kg/m^2^ (IQR 20.28–26.17). Most participants were Thai (70.4%) and nulliparous (78.9%). Thalassemia carriers and individuals with thalassemia disease accounted for 7.8% and 0.6% of the cohort, respectively, while 5.5% had iron deficiency anemia. Baseline characteristics were generally comparable across trimesters. The median gestational age was 11, 16, and 31 weeks in the first, second, and third trimesters, respectively, and the prevalence of iron deficiency anemia was 3.6%, 9.5%, and 3.6%, respectively.

CBC-Hb concentration showed a slight decline during the second trimester, with median/mean values of 12.10 g/dL in the first trimester, 11.50 ± 1.06 g/dL in the second trimester, and 11.90 g/dL in the third trimester. Both tHb and fHb were consistently higher than CBC-Hb values across all trimesters. Thumb SpHb values were 14.40, 14.20, and 14.56 g/dL in the first, second, and third trimesters, as presented in Table 1. Perfusion index (PI) increased progressively with gestational age, rising from 3.10 to 6.70 at the thumb and from 3.80 to 8.08 at the finger between the first and third trimesters. Compared with CBC Hb, SpHb significantly overestimated hemoglobin values at all gestational stages (all *p* < 0.001). The mean bias ranged from 2.15 to 2.80 g/dL for thumb measurements and from 1.85 to 2.37 g/dL for index finger measurements, with a greater overestimation observed at the thumb site, as presented in Table 1.

Overall, while SpHb measurements from both thumb and finger sites correlated with gestational progression and reflected increasing peripheral perfusion, they consistently yielded significantly higher Hb values than standard CBC measurements across all trimesters.

### 3.1. Correlation Between SpHb and CBC Hemoglobin

**Figure 1:**
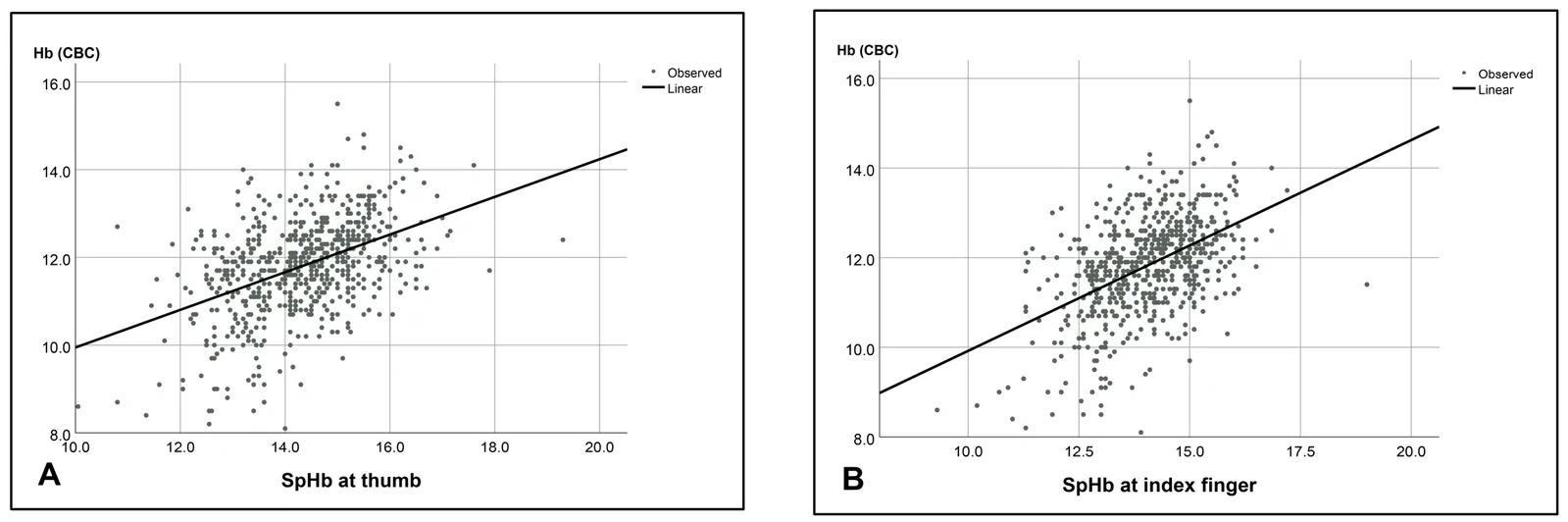
Scatter plots illustrating the linear relationship between SpHb measured at the thumb and CBC-derived Hb (*r*^2^ = 0.197; *p*-value < 0.001) (**A**), and between SpHb measured at the index finger and CBC-derived Hb (*r*^2^ = 0.232; *p*-value < 0.001) (**B**). (SpHb, spectrophotometry hemoglobin; CBC, complete blood count; Hb, hemoglobin).

Pearson correlation analysis demonstrated statistically significant moderate correlations between SpHb and CBC-derived hemoglobin (CBC-Hb) for both thumb SpHb (tHb; *r* = 0.444, *p* < 0.001) and finger SpHb (fHb; *r* = 0.482, *p* < 0.001). In contrast, the correlation between tHb and fHb was strong (*r* = 0.707, *p* < 0.001). Linear regression analysis showed positive linear relationships between both SpHb measurements and CBC-Hb, with coefficients of determination (*R*^2^) of 0.197 for tHb and 0.232 for fHb (Figure 1).

### 3.2. Agreement Between SpHb and CBC Hemoglobin

**Figure 2:**
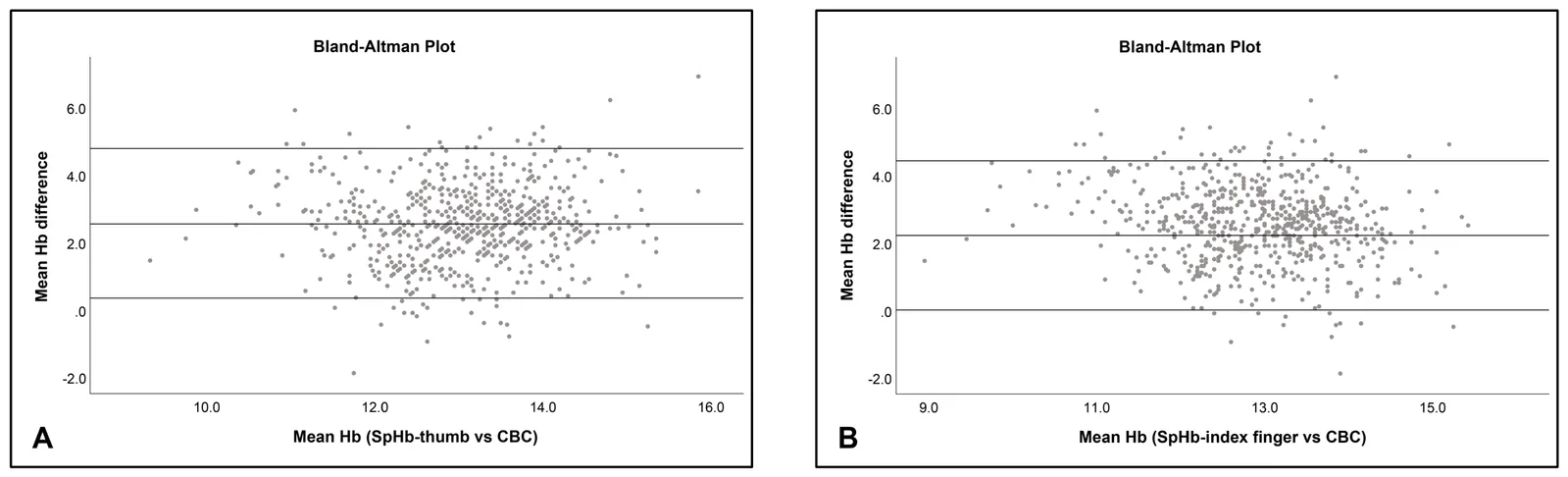
Bland−Altman plots demonstrating agreement between CBC-derived hemoglobin (CBC-Hb) and SpHb measured at the thumb (**A**) and index finger (**B**). Although fewer than 10% of measurements fell outside the 95% limits of agreement, both measurement sites showed substantial positive mean bias.

SpHb measurements obtained from both the thumb (tHb) and finger (fHb) were consistently higher than CBC-derived hemoglobin (CBC-Hb) throughout pregnancy, with mean biases of 2.533 g/dL and 2.221 g/dL, respectively (both one-sample *t*-tests against zero bias, *p* < 0.001). Despite this systematic overestimation, Bland–Altman analysis demonstrated acceptable agreement between SpHb and CBC-Hb, with fewer than 10% of measurements falling outside the 95% limits of agreement with substantial and significant bias, as presented in Figure 2. Intraclass correlation coefficient (ICC) analysis showed poor absolute agreement between SpHb and CBC-Hb. For thumb-derived SpHb (tHb), the ICC for absolute agreement was 0.220, whereas the ICC for consistency was 0.615, indicating moderate consistency despite poor absolute agreement. Similarly, for index finger-derived SpHb (fHb), the ICC for absolute agreement was 0.278, whereas the ICC for consistency was 0.650. These findings suggest that SpHb measurements were relatively precise but lacked accuracy because of systematic measurement bias, as presented in Table 2.

### 3.3. Diagnostic Accuracy for Anemia Detection

The overall prevalence of anemia based on CBC-derived hemoglobin (CBC-Hb) was 14.4% (95/659) across the three trimesters. SpHb demonstrated extremely low sensitivity for detecting anemia at both measurement sites (2.1% for thumb SpHb [tHb] and 3.2% for finger SpHb [fHb]; Table 3). In contrast, specificity was very high (99.8% and 100%, respectively). McNemar’s test showed significant disagreement between anemia classifications based on SpHb and CBC-Hb across all trimesters (all *p* < 0.001).

### 3.4. Potential Confounding Factors on Predicting CBC-Hb

Based on the multivariable regression analyses, t-Hb and f-Hb were significant independent predictors of CBC-Hb in all models. The results indicate that parsimonious models performed similarly to the full models, as presented in Table 4. Across all models, SpHb measured at either the thumb or index finger was the strongest predictor of CBC-Hb. Higher BMI was consistently associated with higher Hb levels, whereas women in the second and third trimesters had significantly lower hemoglobin levels than those in the first trimester. Additional physiological variables (age, oxygen saturation, and pulse rate) provided limited incremental predictive value, particularly for the index-finger model, supporting the use of the more parsimonious models for estimating CBC-Hb. Notably, for the thumb model, perfusion index demonstrated a small but significant negative association in both full and parsimonious models (*p* = 0.024 and 0.041 respectively) while for the index finger model, perfusion index demonstrated no significant impact.

### 3.5. Performance of Adjusted SpHb in Predicting Maternal Anemia

**Figure 3:**
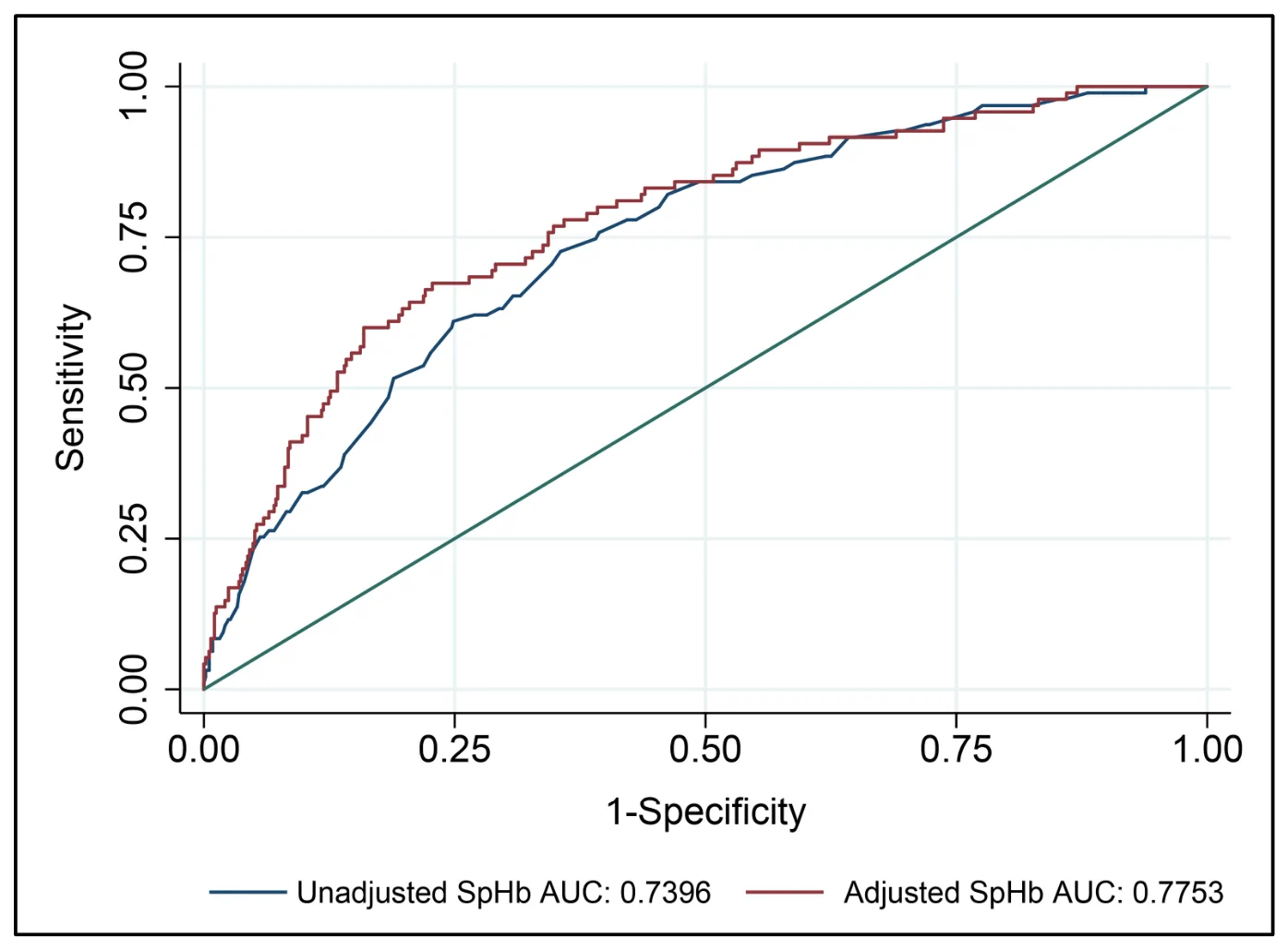
Receiver operating characteristic (ROC) curves demonstrating the diagnostic performance of unadjusted and adjusted SpHb (measured at index fingers) for detecting maternal anemia.

When the predicted CBC-Hb values (representing adjusted SpHb) were used to identify maternal anemia, the adjusted SpHb demonstrated good diagnostic performance for both thumb- and index finger-derived models, with AUCs of 0.745 and 0.775, respectively. These values were slightly, but not significantly, higher than those of the corresponding unadjusted SpHb measurements (AUCs of 0.740 and 0.775, respectively, DeLong test: *p*-value 0.081) (Figure 3). Using the optimal probability cut-off of 0.112, the adjusted SpHb models achieved good diagnostic accuracy, with a sensitivity of approximately 80% and a specificity of approximately 60% for both thumb- and index finger-derived measurements (Table 5).

## 4. Discussion

This study provides several important insights. (1) With the current Masimo Rad-67 calibration, SpHb cannot be used interchangeably with CBC-Hb in Thai pregnant women because systematic overestimation results in very low sensitivity for anemia detection. (2) Although agreement in absolute accuracy was poor, SpHb demonstrated moderate to acceptable precision compared with CBC-Hb. (3) SpHb measurements were influenced by pre-pregnancy BMI, gestational age, and possibly the perfusion index. (4) Unadjusted SpHb showed moderate discrimination for anemia (AUC approximately 0.74), with a trend toward improved performance after adjustment. (5) SpHb measurements obtained from the thumb and index finger were comparable, although values measured at the thumb were modestly higher. Collectively, SpHb showed moderate agreement with CBC-Hb, but its diagnostic performance for maternal anemia was modest in this low-prevalence cohort. Further validation in diverse pregnant populations and clinical settings is needed before broader clinical use, including assessment of whether adjustment for gestational age and maternal BMI improves performance.

The observed overestimation of hemoglobin by the Masimo Rad-67 is consistent with several recent studies but contrasts with earlier reports. Farooq et al. similarly reported poor diagnostic accuracy of SpHb for anemia in pregnant women [15]. However, our sensitivity (2.1–3.2%) was substantially lower than that reported by Caulfield et al. (20%) and Koech et al. (18.66%) [16,20], possibly owing to the lower anemia prevalence in our cohort (11.2–19.1%) and differences in the timing of measurements, which were obtained during routine antenatal care rather than at delivery.

Yoshida et al. reported better agreement between SpHb and laboratory hemoglobin in Japanese pregnant women, although accuracy declined at lower hemoglobin concentrations [21]. Differences from our findings may reflect variations in ethnicity, baseline hemoglobin, anemia prevalence, measurement protocols, device calibration, statistical methods, and study populations, as many previous studies were conducted in non-pregnant or acute-care settings rather than routine antenatal care.

### 4.1. Potential Confounders

The findings were highly consistent between tHb and fHb. In both parsimonious models, SpHb, BMI, and trimester remained independent predictors of CBC-Hb, while additional physiological variables contributed little incremental predictive value. The similarity of regression coefficients and retained predictors across thumb- and index-finger models suggests that the relationship between non-invasive hemoglobin measurement and laboratory hemoglobin is robust regardless of sensor placement, supporting the clinical feasibility of either measurement site.

### 4.2. Correlation and Agreement Between SpHb and CBC-Hb

Correlation and regression analyses demonstrated a positive but weak association between SpHb and CBC-Hb, as reflected by the low correlation coefficient and coefficient of determination (R^2^). ICC analysis further demonstrated poor absolute agreement but acceptable consistency between the two measurement methods. Similarly, Bland–Altman analysis indicated acceptable overall agreement, with most observations falling within the 95% limits of agreement. Nevertheless, SpHb consistently overestimated CBC-Hb by approximately 2.2–2.5 g/dL, resulting in a substantial positive systematic bias. This clinically meaningful magnitude of bias precludes direct interchangeability between SpHb and CBC-Hb for clinical hemoglobin measurement. Although adjustment models improved the diagnostic performance of SpHb for anemia detection, the observed systematic overestimation indicates that further calibration is required before the device can be reliably adopted for clinical use.

### 4.3. Role of Perfusion Index (PI)

Our study demonstrated that PI significantly influenced accuracy of tHb. Overall, PI ≥ 3.0 was associated with greater tHb discrepancy, suggesting that higher pulsatile blood flow may amplify the device’s inherent overestimation bias in pregnant women rather than improve measurement accuracy. Evidence regarding the effect of PI on SpHb performance in pregnant women remains limited. This finding contrasts with Nguyen et al. [18], who reported greater bias at PI < 2 in cardiac surgery patients, indicating that the effect of PI may differ according to the physiological state. Furthermore, the impact of PI was gestational age-dependent, with progressively greater influence in late gestation, likely reflecting the increasing peripheral vasodilation of pregnancy that is not adequately accounted for by the current calibration algorithm.

### 4.4. Clinical Implications

These findings suggest that, at its current calibration, the Masimo Rad-67 is not yet suitable for clinical use. Its implementation should await the development and validation of ethnicity-, pregnancy-, and gestational age-specific calibration algorithms that account for other relevant confounding factors, followed by confirmation of non-inferiority in prospective clinical studies.

### 4.5. Strengths and Limitations

The strengths of this study include the prospective design, large sample size with equal representation across all three trimesters, standardized measurement protocols, use of validated laboratory methods as the reference standard, and comprehensive assessment of factors affecting measurement accuracy including perfusion index. This study was conducted in two different clinical settings (tertiary and secondary care), enhancing generalizability to routine antenatal care in Thailand.

Several limitations should be acknowledged. This single-ethnic group study may have limited generalizability to populations with different ethnic backgrounds, skin pigmentation, or baseline Hb concentrations. Although measurements were performed under standardized conditions, factors affecting pulse CO-oximetry accuracy, including skin pigmentation, ambient temperature, and motion artifacts, were not systematically evaluated. In addition, the findings may not be generalizable to high-risk pregnancies or emergency settings, as this study included only women attending routine antenatal care. Finally, only a single device model (Masimo Rad-67) was evaluated, and performance may differ with other devices or newer technologies, and the performance of the model was analyzed in the same cohort without external validation.

### 4.6. Future Directions

At present, SpHb measurement using the Masimo RAD-67 is not sufficiently reliable for routine clinical application. Both tHb and fHb demonstrated very low sensitivity for detecting anemia, resulting in a substantial proportion of anemic cases being missed. Nevertheless, SpHb showed a moderate linear correlation with CBC-derived hemoglobin (CBC-Hb) and acceptable diagnostic performance based on ROC analysis, suggesting the presence of a systematic measurement bias rather than random error. Notably, diagnostic performance improved considerably after applying optimized cut-off values and adjusting for potential confounding factors, particularly BMI, gestational trimester, and perfusion index. These findings indicate that SpHb has considerable potential for further refinement. Although directly measured SpHb differed significantly from the reference CBC-Hb values, corrected SpHb estimates derived from a multivariable prediction model more closely approximated the true hemoglobin concentration. Therefore, SpHb may become suitable for clinical use once correction algorithms are further optimized and prospective non-inferiority studies demonstrate that the corrected SpHb provides diagnostic performance comparable to that of CBC-Hb. Because the performance of SpHb observed in the present study differed from that reported in studies involving non-pregnant populations and other ethnic groups, future prediction models should be developed and validated specifically for pregnant women within individual ethnic populations. Furthermore, gestational age, represented by categorical trimester in the current analysis, had a significant influence on SpHb measurements. Future studies should therefore incorporate gestational age as a continuous variable rather than a categorical variable to more precisely adjust SpHb values and improve the robustness and predictive performance of the resulting models.

## 5. Conclusions

The Masimo Rad-67 consistently overestimated Hb concentrations in pregnant women across all trimesters. At the manufacturer’s current calibration, this systematic bias resulted in very low sensitivity for anemia screening despite high specificity. Although diagnostic performance improved with optimized cut-offs, SpHb remained only moderately correlated with CBC-Hb, with persistent overestimation. Measurement accuracy was significantly influenced by gestational age, maternal BMI, and perfusion index. Therefore, the Masimo Rad-67 cannot currently be recommended as a standalone screening tool for antenatal anemia. Its clinical utility may be potentially clinically applicable, if confirmed by external validation improved through ethnicity-specific calibration algorithms incorporating gestational age and other relevant confounding factors.

## Figures and Tables

**Table 1:** Comparison of CBC-derived hemoglobin, SpHb, and perfusion index across the three trimesters.

Parameter	First Trimester (*n* = 220)	Second Trimester (*n* = 220)	Third Trimester (*n* = 220)
CBC Hb, median (IQR)	12.10 (11.60–12.80)	11.50 ± 1.06 *	11.90 (11.30–12.40)
Thumb SpHb, median (IQR)	14.40 (13.50–15.20)	14.20 (13.40–14.95)	14.56 ± 1.15 *
Thumb PI, median (IQR)	3.10 (2.10–5)	4.90 (2.70–7.95)	6.70 (3.58–9.60)
Finger SpHb, median (IQR)	14.02 ± 1.04 *	13.85 (13.10–14.80)	14.25 (13.35–14.88)
Finger PI, median (IQR)	3.80 (2.23–6.45)	6 (3.40–8.80)	8.08 (4.98–11.73)
Thumb Hb–CBC Hb	2.15 (*p* < 0.001)	2.67 (*p* < 0.001)	2.80 (*p* < 0.001)
Finger Hb–CBC Hb	1.85 (*p* < 0.001)	2.37 (*p* < 0.001)	2.35 (*p* < 0.001)

* Mean ± SD for normally distributed data. (SpHb, spectrophotometry hemoglobin; CBC, complete blood count; Hb, hemoglobin; PI, perfusion index; IQR, interquartile range.)

**Table 2:** Intraclass correlation coefficient for agreement between SpHb and CBC-Hb.

	Intraclass Correlation (95% CI)			*p*-Value
*SpHb measured at thumb*				
Average Measures (absolute type)	0.220	−0.141	0.529	<0.001
Average Measures (consistency type)	0.615	0.551	0.670	<0.001
*SpHb measured at index finger*				
Average Measures	0.278	−0.166	0.599	<0.001
Average Measures	0.650	0.593	0.700	<0.001

**Table 3:** Diagnostic accuracy of SpHb for detecting anemia using the same trimester-specific hemoglobin criteria as CBC-Hb.

		95% Confidence Interval
*SpHb measured at the thumb*		
Prevalence	14.2%	11.7–17.1%
Sensitivity	2.1%	0.3–7.4%
Specificity	99.8%	99.0–100.0%
Positive predictive value	66.7%	9.4–99.2%
Negative predictive value	86.0%	83.1–88.6%
Positive likelihood ratio	12.06	1.10–131.73
Negative likelihood ratio	0.98	0.95–1.01
*SpHb measured at the index finger*		
Prevalence	14.2%	11.7–17.1%
Sensitivity	3.2%	0.7–9.0%
Specificity	100.0%	99.4–100.0%
Positive predictive value	100.0%	29.2–100.0%
Negative predictive value	86.2%	83.3–88.7%
Positive likelihood ratio	-	-
Negative likelihood ratio	0.97	0.93–1.00

**Table 4:** Comparison of full and parsimonious multivariable regression models using thumb- and index finger-derived non-invasive hemoglobin (SpHb) to predict CBC hemoglobin.

CBC-Hb	Coefficient	Standard Error	z- Statistics	*p*- Value	95% CI Lower	95% CI Upper
**SpHb (thumb)**						
*Full model*						
SpHb (thumb)	0.42370	0.03366	12.59	0.000	0.35772	0.48967
Age	0.00729	0.00647	1.13	0.260	−0.00538	0.01996
BMI	0.02903	0.00730	3.97	0.000	0.01472	0.04335
Trimester: (first = base)						
second	−0.61164	0.09089	−6.73	0.000	−0.78977	−0.43350
third	−0.48583	0.09747	−4.98	0.000	−0.67687	−0.29480
Perfusion index	−0.01143	0.00505	−2.26	0.024	−0.02133	−0.00152
O2 saturation	−0.05620	0.04372	−1.29	0.199	−0.14189	0.02948
Pulse rate	−0.00489	0.00315	−1.55	0.120	−0.01106	0.00128
Constant	11.22828	4.49696	2.50	0.013	2.41439	20.04216
Parsimonious model *						
SpHb (thumb)	0.42408	0.03325	12.75	0.000	0.35890	0.48925
BMI	0.03016	0.00691	4.36	0.000	0.01661	0.04371
Trimester: (first = base)						
second	−0.59163	0.08941	−6.62	0.000	−0.76686	−0.41639
third	−0.46142	0.09048	−5.10	0.000	−0.63876	−0.28409
Perfusion index	−0.01011	0.00495	−2.04	0.041	−0.01982	−0.00040
Constant	5.42113	0.48497	11.18	0.000	4.47060	6.37166
**SpHb (index finger)**						
*Full model*						
SpHb (index finger)	0.45021	0.03252	13.85	0.000	0.38648	0.51395
Age	0.00335	0.00648	0.52	0.605	−0.00935	0.01605
BMI	0.03033	0.00717	4.23	0.000	0.01628	0.04437
Trimester (first = base)						
second	−0.61198	0.08963	−6.83	0.000	−0.78764	−0.43632
third	−0.45202	0.09675	−4.67	0.000	−0.64164	−0.26240
Perfusion index	−0.00049	0.00113	−0.43	0.665	−0.00271	0.00173
O2 saturation	−0.02364	0.04359	−0.54	0.588	−0.10908	0.06179
Pulse rate	−0.00199	0.00332	−0.60	0.548	−0.00850	0.00451
Constant	7.54422	4.48968	1.68	0.093	−1.25540	16.34384
*Parsimonious model*						
SpHb (index finger)	0.44811	0.03211	13.96	0.000	0.38518	0.51104
BMI	0.03062	0.00674	4.54	0.000	0.01740	0.04384
Trimester (first = base)						
second	−0.59877	0.08717	−6.87	0.000	−0.76962	−0.42793
third	−0.42908	0.08748	−4.90	0.000	−0.60054	−0.25762
Constant	5.14273	0.46555	11.05	0.000	4.23028	6.05518

* The parsimonious models retained only variables that remained independently associated with CBC-Hb.

**Table 5:** Diagnostic accuracy of adjusted SpHb for detecting maternal anemia, using trimester-specific CBC-Hb hemoglobin thresholds as the reference standard.

		95% Confidence Interval
*SpHb measured at the thumb*		
Prevalence	14.30%	11.7–17.2%
Sensitivity	80.00%	70.5–87.5%
Specificity	59.40%	55.2–63.4%
Positive predictive value	24.70%	20.0–29.9%
Negative predictive value	94.70%	91.8–96.8%
Positive likelihood ratio	1.97	1.71–2.27
Negative likelihood ratio	0.34	0.22–0.51
*SpHb measured at the index finger*		
Prevalence	14.30%	11.7–17.2%
Sensitivity	80.00%	70.5–87.5%
Specificity	60.80%	56.6–64.8%
Positive predictive value	25.30%	20.5–30.7%
Negative predictive value	94.80%	92.0–96.8%
Positive likelihood ratio	2.04	1.77–2.35
Negative likelihood ratio	0.33	0.22–0.49

## Data Availability

The raw data supporting the conclusions of this article will be made available by the authors on request.
